# Hydrolytic Profile of the Culturable Gut Bacterial Community Associated With *Hermetia illucens*

**DOI:** 10.3389/fmicb.2020.01965

**Published:** 2020-08-12

**Authors:** Matteo Callegari, Costanza Jucker, Marco Fusi, Maria Giovanna Leonardi, Daniele Daffonchio, Sara Borin, Sara Savoldelli, Elena Crotti

**Affiliations:** ^1^Dipartimento di Scienze per gli Alimenti, la Nutrizione e l’Ambiente (DeFENS), Università degli Studi di Milano, Milan, Italy; ^2^Red Sea Research Center (RSRC), King Abdullah University of Science and Technology (KAUST), Thuwal, Saudi Arabia; ^3^School of Applied Sciences, Edinburgh Napier University, Edinburgh, United Kingdom

**Keywords:** black soldier fly, waste valorization, nutrient recycling, larval weight, pupal weight, bacterial isolation, probiotics

## Abstract

Larvae of the black soldier fly (BSF) *Hermetia illucens* (L.) convert organic waste into high valuable insect biomass that can be used as alternative protein source for animal nutrition or as feedstock for biodiesel production. Since insect biology and physiology are influenced by the gut microbiome, knowledge about the functional role of BSF-associated microorganisms could be exploited to enhance the insect performance and growth. Although an increasing number of culture-independent studies are unveiling the microbiota structure and composition of the BSF gut microbiota, a knowledge gap remains on the experimental validation of the contribution of the microorganisms to the insect growth and development. We aimed at assessing if BSF gut-associated bacteria potentially involved in the breakdown of diet components are able to improve host nutrition. A total of 193 bacterial strains were obtained from guts of BSF larvae reared on a nutritious diet using selective and enrichment media. Most of the bacterial isolates are typically found in the insect gut, with major representatives belonging to the Gammaproteobacteria and Bacilli classes. The hydrolytic profile of the bacterial collection was assessed on compounds typically present in the diet. Finally, we tested the hypothesis that the addition to a nutritionally poor diet of the two isolates *Bacillus licheniformis* HI169 and *Stenotrophomonas maltophilia* HI121, selected for their complementary metabolic activities, could enhance BSF growth. *B. licheniformis* HI169 positively influenced the larval final weight and growth rate when compared to the control. Conversely, the addition of *S. maltophilia* HI121 to the nutritionally poor diet did not result in a growth enhancement in terms of larval weight and pupal weight and length in comparison to the control, whereas the combination of the two strains positively affected the larval final weight and the pupal weight and length. In conclusion, we isolated BSF-associated bacterial strains with potential positive properties for the host nutrition and we showed that selected isolates may enhance BSF growth, suggesting the importance to evaluate the effect of the bacterial administration on the insect performance.

## Introduction

A growing body of evidence is supporting the gut microbiota as a component of paramount importance for the host physiology, development and health (as reviewed, among others, by Sommer and Bäckhed, 2013, and Schwarzer et al., 2018). Metataxonomic sequencing (primarily targeting the bacterial 16S rRNA gene or the fungal Internal Transcribed Spacer, ITS) is the most commonly used culture-independent method to characterize the host-associated microbial communities at the taxonomic, structural and network levels. On the other hand, functional profiles of the host microbial partners are typically investigated by metagenomic sequencing, which requires a deep sequencing coverage to precisely assign functions to different host-associated strains. Nevertheless, these approaches cannot disentangle the contributions of closely related but functionally different strains (Forster et al., 2019), with few exceptions reported on honeybees which show a simple and conserved microbial community composition (Engel et al., 2012; Ellegard and Engel, 2016, 2019). Functional properties of closely related strains in the same species can differ substantially (Barbato et al., 2019) and their metabolic potential may not be easily revealed by culture-independent methods (Prakash et al., 2013). Microbial cultures remain the gold standard to experimentally validate the microbial role and to obtain reference genome sequences that help in elucidating the functional roles of microorganisms (Forster et al., 2019), highlighting the importance of integrated “culturomics” approaches (Lagier et al., 2016). Additionally, microbial cultivation is pivotal for selecting and validating beneficial probiotic strains capable, when administered to the host, to promote food digestion, enhance growth or protect from pathogens (Prakash et al., 2013). However, culturable strains represent only a small part of the microbiome associated to a specific host or habitat: it has been generally estimated that only 1–10% of the bacterial diversity has been cultivated (Prakash et al., 2013) and that this value depends on the considered environment (Lagkouvardos et al., 2017). Thus, in regard to host-microbe interactions, it is, however, noteworthy to point out that also the unculturable fraction may contain important symbionts that cannot be exploited just because they are not culturable (Hosokawa et al., 2016; Ankrah et al., 2018).

The black soldier fly (BSF) *Hermetia illucens* (L.) (Diptera: Stratiomyidae) is a promising candidate for waste management, feed production or biodiesel conversion (van Huis, 2013; Makkar et al., 2014). It is native of the tropical and warm temperate zone of America and it is currently present in many countries around the world. Adults are considered non-pest insects that can survive without feeding, but their lifespan can be significantly extended in the presence of a sucrose solution or water (Lupi et al., 2019). Saprophagous larvae can consume a wide range of organic materials, ranging from food waste to animal remains and manures, converting them into high value biomass rich in proteins and fats (Sheppard et al., 1994; Makkar et al., 2014; Nguyen et al., 2015; Bava et al., 2019; Jucker et al., 2020). BSF has been shown to decrease *Escherichia coli* and *Salmonella* spp. population loads in manures (Liu et al., 2008; Lalander et al., 2015), probably due to the abundance of different antimicrobial peptides (AMPs) that it can produce (Vogel et al., 2018). Since it has been estimated that the food wastage (a term that includes both food waste and food lost) accounts approximately for one third of all the food produced for human consumption (FAO, 2013), BSF could be used for organic waste reduction and valorization, converting it into biomass with a final added value as feed, food or fuel (van Huis, 2013; Nyakeri et al., 2017; Chia et al., 2018; Onsongo et al., 2018; Shumo et al., 2018; Cappellozza et al., 2019). Under this perspective and considering the increasing exploitation of *H. illucens* for bio-waste disposal and its conversion in animal feed, elimination of toxic and anti-nutritional compounds (i.e., that can reduce the availability of nutrients such as phytate) present in the insect diet source could be also an important aspect to take into account in order to improve the insect growth.

In recent years, BSF microbiota has been investigated considering different host developmental stages and feeding conditions. The studies have highlighted that both the diet source and life stage directly influenced the microbiota diversity (Jeon et al., 2011; Zheng et al., 2013b; Varotto Boccazzi et al., 2017; De Smet et al., 2018; Jiang et al., 2019; Wynants et al., 2019) and it has been recently shown that the bacterial communities varied in density and phylogenetic composition along the anterior, middle and posterior portions of the midgut (Bruno et al., 2019).

As potential probiotics BSF-associated microorganisms could be exploited to sustain the insect performance (Crotti et al., 2012). For instance, larval development on poultry manure has been shown to benefit by supplementation with *Bacillus subtilis* isolated from BSF both in term of larval weight increase and developmental time reduction (Yu et al., 2011). The strategy to assess the role of specific isolates as beneficial probiotics characterizes their phenotypic properties *in vitro* to select potentially suitable strains and tests their effectiveness in promoting larval weight, growth rate and adult survival along the insect cycle, upon supplementation in the host diet (Prosdocimi et al., 2015). In this work we implemented such strategy on a large collection of bacterial isolates from the gut of BSF larvae reared on a nutritionally complete diet (standard diet, SD). The isolates were identified and functionally screened *in vitro* for potentially valuable nutritional properties. Two selected isolates with high probiotic potential were then tested *in vivo* for their potential contribution to BSF development, by their administration to larvae reared on a nutritionally poor diet (fruit diet, FD). Specifically, we have screened the isolates for the ability to degrade organic waste polymers (such as cellulose, starch and pectin), produce exopolysaccharides (EPS) potentially useful for adhesion to the host epithelium and for the potential to contribute to the host nitrogen metabolism and phosphorous recycling.

## Materials and Methods

### Insect Colony

*Hermetia illucens* was reared at the entomological facility of the University of Milan, Italy (Jucker et al., 2017). BSF larvae were fed *ad libitum* on a nutritionally complete standard diet (SD), composed of wheat germ 50%, alfalfa 30%, corn flour 20%, to which is added an equal volume of water according to Hogsette (1992), under controlled conditions of 25°C and 60–65% relative humidity (RH).

### Larval Gut Dissection and Bacterial Isolation

Guts from four larvae (with a weight between 0.14 and 0.16 g), reared on standard diet (SD), were used for bacterial isolation. All the isolation procedures were performed in aerobic conditions. Insects were dissected close to the flame of a Bunsen burner using sterile forceps and needles, after a step of external surface sterilization that was done to exclude the epibiont microorganisms. Larvae were first washed in 50 ml tubes according to the following protocol: sodium dodecyl sulphate (SDS) 0.1% for 5 min, sodium hypochlorite 1% for 3 min, ethanol 70% for 1 min three times and finally rinsed five times in sterilized distilled water. The water from the last rinse was plated on agarized Nutrient Broth (NB) and incubated at 30°C for 72 h in order to evaluate the sterilization efficiency. Once dissected in sterile phosphate-buffered saline (PBS), guts were homogenized using sterile plastic pestles in 900 μl of 0.9% NaCl, which were then used to inoculate liquid enrichment media in order to select uricolytic and cellulolytic strains. Specifically, we used 3 different enrichment media: (i) enrichment uric acid medium [0.8% uric acid; 0.05% KH_2_PO_4_; 0.2% K_2_HPO_4_; 0.01% NaCl; 0.01% MgSO_4_⋅7H_2_O; 0.01% CaCl_2_⋅2H_2_O; pH 7.0 (Ghosh and Sarkar, 2014)]; (ii) enrichment FP medium (0.25%NaNO_3_, 0.2% KH_2_PO_4_, 0.02% MgSO_4_⋅7H_2_O, 0.02% NaCl, 0.01% CaCl_2_⋅6H_2_O, and Filter paper (FP) - Whatman no. 1, two disks of 2.00 cm^2^ per 30 ml); and (iii) enrichment CMC (carboxymethylcellulose) medium [0.25% NaNO_3_, 0.2% KH_2_PO_4_, 0.02% MgSO_4_⋅7H_2_O, 0.02% NaCl, 0.01% CaCl_2_⋅6H_2_O, and 0.2% CMC (Gupta et al., 2012)]. Liquid media were then incubated at 30°C in shaking conditions. Subcultures were made every 7 days, for three times, and finally plated onto Basal-Trace (BT) solid medium (0.3% uric acid, 0.2% K_2_HPO_4_, 0.05% KH_2_PO_4_, 0.01% MgSO_4_⋅7H_2_O, 0.01% NaCl, 0.01% CaCl_2_, 1% (v/v) trace element solution (5.0% FeSO_4_⋅7H_2_O, 5% CuSO_4_⋅7H_2_O), pH 7.0, agar 1.5%) in case of cultures from Enrichment Uric Acid medium (Ghosh and Sarkar, 2014), whereas FP and CMC liquid media were plated on CMC agar plates (Gupta et al., 2012). In addition, serial dilutions (0.1 ml) of the gut homogenates were spread on the surface of different types of plates: (i) basal medium (0.1% [NH_4_]NO_3_; 0.1% yeast extract; 50 ml standard salt solution; 1 ml trace element solution and 1.5% agar and final pH 7.0) added with 0.5% Avicel (Sigma-Aldrich) or 0.5% CMC with 0.1% Congo-red (Sigma-Aldrich) for differential isolation of exo- or endo-cellulolytic, respectively (Ventorino et al., 2015); (ii) casein agar (1.5% peptone from casein; 0.5% soy peptone; 0.5% NaCl; 1.5% agar; pH 7.3 ± 0.2); (iii) pectin agar (0.4% [NH_4_]SO_4_, 0.01% NaCl; 0.01% MgSO_4_⋅7H_2_O, 0.01% CaCl_2_⋅2H_2_O, 0.05% yeast extract, 0.0052% Fe(III)-citrate, 500 ml potassium phosphate buffer (50% K_2_HPO_4_ 1M + 50% KH_2_PO_4_ 1M), 0.5% pectin, agar 1.5%, pH adjusted to 7.0); (iv) chitin agar (0.25% NaNO_3_; 0.2% K_2_HPO_4_; 0.02% MgSO_4_⋅7H_2_O; 0.02% NaCl; 0.01% CaCl_2_⋅2H_2_O; 0.5% chitin from crab shells; 1.5% agar pH 6.8–7.2); (v) nutrient agar-uric acid (NA-UA) medium [0.5% gelatin peptone, 0.3% beef extract, 0.2% NaCl, 0.3% uric acid, pH 7.0, and agar 1.5% (Ghosh and Sarkar, 2014)]. When colonies appeared on the agar plates, they were picked up and streaked on the same media used for the isolation three times to ensure purity. The bacterial collection was then conserved in 25% glycerol solution at −80°C.

### Bacterial Identification

Total DNA from each isolate was extracted by boiling lysis (Ferjani et al., 2015) or using a phenol-chloroform DNA extraction based protocol (Sambrook et al., 1989) in case of 16S rRNA PCR amplification failure on bacterial DNA extracted according to boiling lysis. The bacterial collection was dereplicated by ITS-PCR fingerprinting using the primer pair ITS-F (5′-GTC GTA ACA AGG TAG CCG TA-3′) and ITS-Reub (5′-GCC AAG GCA TCC ACC-3′) (Mapelli et al., 2013; Soldan et al., 2019). For each ITS group one/two candidates were selected and the 16S rRNA gene was amplified using the primers 27F (5′-AGA GTT TGA TCM TGG CTC AG -3′) and 1492R (5′- CTA CGG CTA CCT TGT TAC GA -3′) (Mapelli et al., 2013; Soldan et al., 2019). PCR fragments were partially sequenced at Macrogen (South Korea) and sequences were then aligned against the EzBioCloud database (Yoon et al., 2017). Sequences were deposited at the European Nucleotide Archive under the accession number PRJEB30516.

### Screening of Metabolic Activities of Bacterial Isolates

For amylase-screening, bacterial cultures were spotted onto NB agar plate supplemented with starch (1%) and then incubated at 30°C for 48 h. After incubation, plates were flooded with 1% Lugol’s iodine solution (Jacob and Gerstein, 1960) to identify extra-cellular amylase activity.

Cellulase-screening was performed as described by Ventorino et al. (2015) using a medium containing 0.1% [NH_4_]NO_3_, 0.1% yeast extract, 50 ml standard salt solution, 1 ml trace element solution (0.01% H_3_BO_3_, 0.012% MnSO_4_ H_2_O, 0.125% ZnSO_4_ 7H_2_O, 0.078% CuSO_4_ 5H_2_O, 0.01% MoO_3_), 0.5% CMC, 0.1% Congo red (Sigma-Aldrich), and 1.5% agar at pH 7. Five microliters of liquid bacterial cultures grown overnight in shaking conditions at 30°C were spotted on plates and incubated at 30°C for 4 days. After incubation, strains with cellulolytic activity showed clear halo zones around the colonies.

Pectinase-screening medium contained 0.67% Yeast Nitrogen Base, 1.0% pectin, and 1.5% agar at pH 7.0 ± 0.2 (Park et al., 2007). The plates were then treated with 1% n-hexadecyltrimethylammonium bromide solution (CTAB) and pectin degradation was assessed through the observation of a clear halo around the colonies.

Esterase activity was evaluated using a medium composed of 1.0% peptone, 0.5% NaCl, 0.01% CaCl_2_⋅2H_2_O, 1 ml tween 80, and 2.0% agar at pH 7.4 ± 0.2 (modified from Mazzucotelli et al., 2013). A white precipitate formation around the colonies, resulting from the deposition of crystals of calcium salt, indicated the solubilization of fatty acids due to the esterase activity.

Esterase/lipase activity was detected on tributyrin agar medium which contained 0.8% NB, 10 ml tributyrin, 4 ml tween 20 and 1.5% agar at pH 7.5 ± 0.2. Tributyrin agar plates with spotted isolates were incubated at 30°C for 72 h. The clear zone of hydrolysis is indicative of either esterase and/or lipase activity according to Gupta et al. (2003).

True lipase activity was screened by rhodamine oil agar (ROA) medium containing 0.8% NB, 0.4% NaCl, 3.125% olive oil, 10 ml rhodamine B (1 mg/mL solution), and 2% agar at pH 7, following the protocol described by Kumar et al. (2012). After incubation at 37°C for 48 h, positive strains were identified by the formation of orange fluorescent halos around bacterial colonies under UV light.

Extracellular protease activity was assessed using milk agar medium composed of 0.5% pancreatic digest of casein, 0.1% glucose, 0.25% yeast extract, 3.5% skim powder milk and 1.5% agar (modified from Jeon et al., 2011). Plates were examined after 72 h of incubation at 30°C. The appearance of a clear zone around spotted isolates indicated the production of extracellular protease.

Ammonia production was evaluated as described by Cappuccino and Sherman (1992). Briefly, after an overnight incubation at 30°C in TSB in shaking condition, 500 μl of bacterial culture were inoculated in 10 ml of peptone water and incubated at 30°C for 4 days. Then, 1 ml of Nessler reagent was added to each culture: the development of orange color indicated the strain ability to produce ammonia. Uninoculated medium developed a green color, as well as bacteria without ammonia production ability.

To detect urea degradation, isolates were inoculated in tryptic soy broth (TSB) liquid medium and incubated overnight at 30°C in shacking condition; 0.5 ml of cultures were then transferred in 1.5 ml tubes and washed twice with 0.9% NaCl (5 min, 4500 rpm, room temperature) to remove the residual growing medium. Pellets were resuspended with 470 μl of solution B (0.1% KH_2_PO_4_, 0.1% K_2_HPO_4_, 0.5% NaCl, 0.013% NiCl_2_, 1 mL phenol red 0.2%, and 100 mL dH_2_O) and 30 μl of solution A (2 g urea, 2 mL ethanol and 4 mL dH_2_O) and incubated at 30°C for 1–2 h. Color was then checked: positive strains showed a color change from yellow to bright pink (modified from Mora et al., 2002).

Uric acid breakdown was screened observing the formation of clear haloes around the isolates spotted onto NB-UA plates (0.8% NB, 0.5% uric acid, and 1.5% agar), incubated for 48 h at 30°C (modified from Morales-Jiménez et al., 2013).

Phytase-screening medium (PSM) contained 1% glucose, 0.4% Na-phytate, 0.2% CaCl_2_, 0.5% NH_4_NO_3_, 0.05% KCl, 0.05% MgSO_4_⋅7H_2_O, 0.001% FeSO_4_⋅7H_2_O, 0.001% MnSO_4_⋅H_2_O, and 1.5% agar at pH 7. Degradation of Na-phytate was evaluated after incubation at 30°C for 4 days. The presence of clear zones around the isolates spotted on plates was considered as indication of phytate degradation (Jorquera et al., 2008).

Exopolysaccharides (EPS) production was estimated according to Santaella et al. (2008) using a RCV medium modified with the addition of sucrose (2%). Bacterial strains were streaked on agar plates of RCV medium and, after 5 days of incubation at 30°C, colonies showing translucent and mucoid growth were considered positive for EPS production.

All the bacterial screenings were performed in aerobic conditions.

### Bacterial Administration to the Insect Diet

*Bacillus licheniformis* HI169 and *Stenotrophomonas maltophilia* HI121 cells were administered to *H. illucens*, singularly or in mix. The laboratory strain *Escherichia coli* DH5α pKan(DsRed) was used in the trials as a control strain, outsider of the BSF commensal community (Crotti et al., 2009). Strains HI169 and HI121 were inoculated in tryptic soy broth medium (TSB medium) and cultured overnight at 30°C, whereas *E. coli* DH5α pKan(DsRed) was inoculated in Luria Bertani medium (LB medium) and cultured overnight at 37°C. The following day, 5 ml of the cultures were inoculated in 100 ml of the proper growth media and incubated for 24 h. After growth, cells were centrifuged at 3000 rpm for 15 min at 4°C, the supernatants were discarded and the pellets were washed three times with saline (NaCl 0.9%) in order to remove the spent medium. The collected cells were properly diluted to a final concentration of 10^8^ cfu/ml in saline.

In order to evaluate any possible antagonistic interaction between the strains, *B. licheniformis* HI169 and *S. maltophilia* HI121 were co-cultured on the same plate. Briefly, one strain was inoculated with one single streak in the middle of a TSA plate and incubated for 48 h at 30°C. Then, three streaks of the second strain were perpendicularly carried out close to the previous one. Three replicated plates were prepared for each strain. Co-cultures were incubated at 30°C and checked after 24 and 48 h to evidence the strains’ growth inhibition.

A nutritionally poor fruit diet composed of apple 1/3, pear 1/3 and orange 1/3 (FD, Jucker et al., 2017) was selected to evaluate the effect of bacterial administration on larval growth performance. After eclosion, larvae of BSF were fed on the FD diet for nine days until handling. Each treatment had three replicates set up in three 10.5 × 5 cm plastic containers with ∼ 60 g each of the FD fruit-based diet. Each replicate was inoculated with 10 ml of NaCl 0.9% containing 10^8^ cfu/ml of i) *E. coli* DH5α pKan(DsRed); ii) *S. maltophilia* HI121; iii) *B. licheniformis* HI169; and iv) *S. maltophilia* HI121 and *B. licheniformis* HI169 (in the latter case we considered a final concentration of 10^8^ cfu/ml for the two strains). Sterile saline not treated with bacteria served as negative control. One hundred and fifty 9-day-old BSF larvae were then added into each container, covered with cardboard breathable caps, and stored in the climate chamber under controlled conditions (25°C, 60–65% RH). Ten larvae were randomly selected and weighed every 2/3 days with an analytical balance (SARTORIUS CP64). Final larval weight corresponded to the mean weight of 10 larvae at the moment (day) of the appearance of the first prepupa in each replicate. Time from egg eclosion to the appearance of the first prepupa in each container was recorded. All prepupae were removed from the container at the moment of appearance and counted. Observations continued until all larvae had entered the pupal stage or died.

### Statistical Analysis

To assess the differences of insect performance among the treatments (our explanatory categorical variable; levels: Control, *E. coli* DH5α pKan(DsRed), HI121, HI169, and HI121 + HI169) we measured, as continuous response variables, larval growth, larval final weight, number of prepupae, pupal weight, and pupal length. For larval growth and the appearance of number of prepupae, we tested such differences using a generalize additive model statistic (GAM, package mgcv in ‘r’; Wood, 2001), while for the larval final weight and the pupal weight and length we performed a linear mixed model where we controlled the factor batch (using the package lmerTest in ‘r’; Kuznetsova et al., 2015). For the ANOVA analysis we performed a pair-wise comparison using Tukey HSD test. All statistical analyses were carried out in R (R Core Team, 2018).

## Results

### Bacterial Isolation From BFS Larvae Gut

**FIGURE 1:**
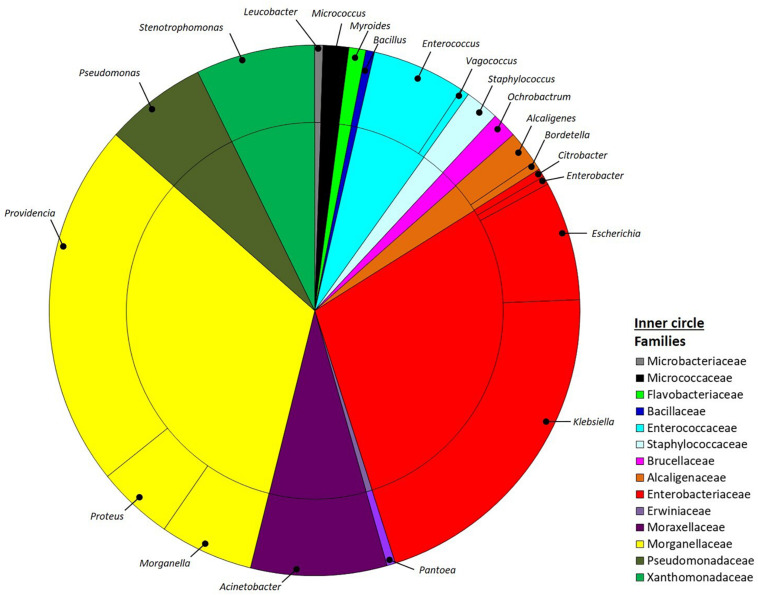
Pie-charts representing the relative culturable bacterial diversity associated to *H. illucens* larvae. Bacterial diversity is expressed at the genus (outer circle) and family (inner circle) levels.

Bacteria were isolated from the guts of four BSF larvae reared on SD using different media specifically selected to obtain isolates with properties potentially relevant for the host nutrition. Upon appearance of colonies on the plates, the isolates were selected according to their morphology and then purified, establishing a collection of 193 pure cultures. After ITS-dereplication, we identified 77 ITS groups that were assigned to four phyla, namely Proteobacteria, Firmicutes, Actinobacteria and Bacteroidetes by 16S rRNA gene partial sequencing. Proteobacteria included 88% of all the total isolates and were assigned to the Alpha, Beta and Gamma-classes (2, 3 and 84%, respectively). Firmicutes isolates (9%) were in the class of Bacilli, whereas Actinobacteria and Bacteroidetes accounted for 2% and 1%, respectively (Figure 1). Within Proteobacteria the most abundant family was Morganellaceae (33%), followed by Enterobacteriaceae (29%), Moraxellaceae (8%), Xanthomonadaceae (7%), Pseudomonadaceae (6%), Alcaligenaceae (3%) Brucellaceae (2%) and Erwiniaceae (1%). Firmicutes were represented by Enterococcaceae (6%), Staphylococcaceae (2%) and Bacillaceae (1%) families. Flavobacteriaceae (1%) was the only family within the phylum of Bacteroidetes, whereas Actinobacteria were divided into Micrococcaceae (2%) and Microbacteriaceae (1%).

Within the entire collection, the most represented genera were *Providencia* (22%) and *Morganella* (6%) in the family Morganellaceae, *Klebsiella* (21%) and *Escherichia* (7%) within the family of Enterobacteriaceae, *Acinetobacter* (8%) in Moraxellaceae family, *Stenotrophomonas* (7%) in the Xanthomonadaceae family, *Pseudomonas* (6) in the Pseudomonadaceae family, and *Enterococcus* (6%) within Enterococcaceae family (Figure 1).

### Hydrolytic Profiles and EPS Production

**FIGURE 2:**
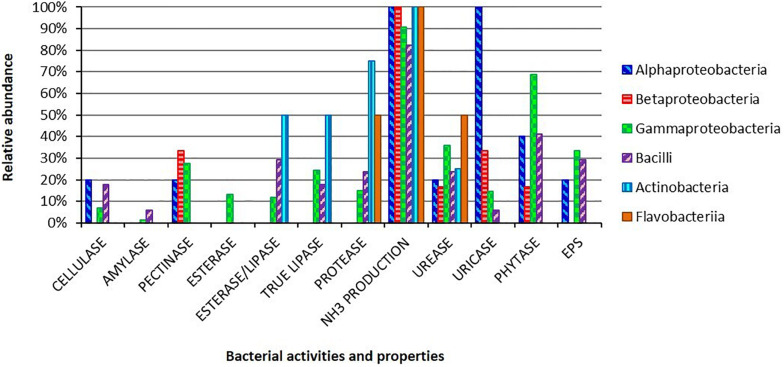
Metabolic activities and EPS production ability for the bacterial isolates obtained from the gut of *H. illucens* larvae. Bars indicate the percentages of each bacterial class in respect to the analyzed hydrolytic activities and EPS production.

All the 193 isolates were screened to characterize the potential contribution of the bacterial partners to the host carbon and nitrogen uptake, as well as to investigate the bacterial ability to adhere to the gut epithelium through the production of adhesive substances, i.e., EPS (Figure 2 and Supplementary Table 1).

Regarding polysaccharide degradation, we found that 15 out of a total of 193 isolates were able to degrade cellulose, 3 were amylolytic, while 47 were pectinolytic bacteria. Lipid degradation ability was present among the isolates: 21 of them were able to degrade Tween 80, 26 to utilize tributyrin and 44 were positive for the true-lipase assay on olive oil plates. Considering nitrogen diet compounds, 175 isolates, 89% of the strain collection, were able to produce ammonia from peptides, and 32 isolates could degrade proteins. The potential capacity to recycle nitrogen from insect metabolic waste compounds was identified in 63 and 31 isolates that resulted positive for the degradation of urea and uric acid, respectively. We also focused our attention on the presence of phytase activity in the bacterial collection, which could release bioavailable phosphate from diet components: we found that 119 strains were positive to the phytate-degradation screening.

The hydrolytic abilities were widespread in our collection. Some activities were specific for some taxonomical groups, i.e., pectinolytic activity for *Klebsiella* spp. and *Stenotrophomonas* spp. strains (Supplementary Table 1). Moreover, several strains showed multiple hydrolytic abilities: 31% of the isolates showed ≥ 4 activities and, among these, 13 strains showed a multi-activity profile resulting positive for 5 or 6 activities out of the 11 that we performed (Supplementary Table 1). Finally, we investigated the EPS production ability obtaining 59 positive strains (30% of the collection; Figure 2).

We pinpoint that not all the bacterial isolates obtained from cultures enriched for a specific degrading activity showed the expected enzymatic activity when tested using specific plate-based assays (Supplementary Table 1). This is probably due to the leaking of organic matter from the original homogenates during dilution procedure/enrichment phases or to the presence of companion strains in the enrichment cultures able to sustain the growth of the non-hydrolytic ones.

### Effect of Bacterial Strains Supplementation on Larval Development

**FIGURE 3:**
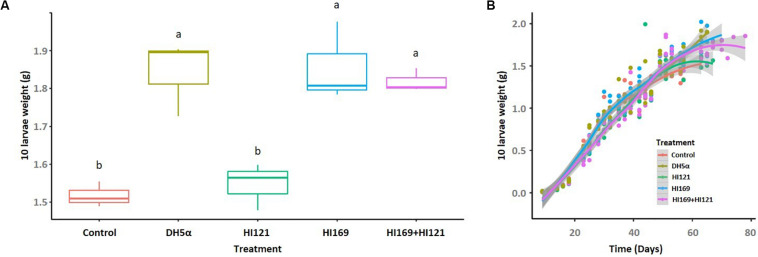
Larval final weight and growth rate following the bacterial administration. **(A)** Final weight of 10 larvae, reported in grams, are shown for the different treatments. **(B)** Growth rate of 10 BSF larvae reared on non-sterile fruit-based diet with or without the selected bacterial strains. The horizontal axis indicates the time (days), while the vertical axis reports the weight of 10 larvae (grams). Control: non-sterile diet without any selected strains; DH5α: larvae fed with the outsider strain of *E. coli* DH5α pKan(DsRed); HI121: larvae added with *S. maltophilia* strain HI121; HI169: larvae supplemented with *B. licheniformis* strain HI169; HI169 + HI121 larvae fed with the two selected strains.

**FIGURE 4:**
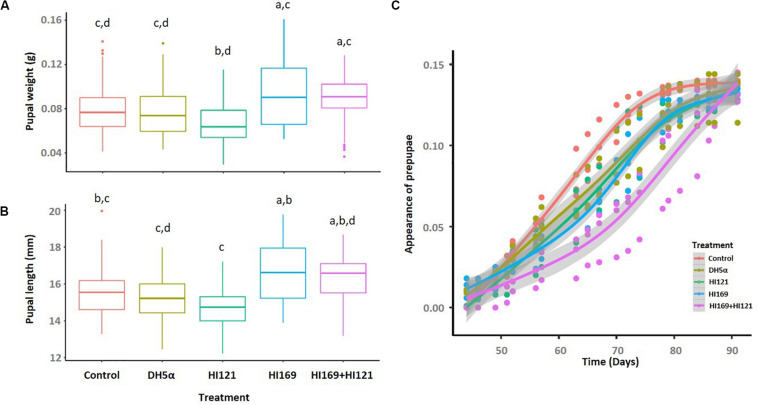
Pupal weight, length and appearance following the bacterial administration. **(A)** Pupal weight and **(B)** length are depicted for the different treatments. **(C)** The prepupal appearance is reported as the progressive average percentages within the population of each treatment. Control: non-sterile diet without any selected strains; DH5α: larvae fed with the outsider strain of *E. coli* DH5α pKan(DsRed); HI121: larvae added with *S. maltophilia* strain HI121; HI169: larvae supplemented with *B. licheniformis* strain HI169; HI169 + HI121 larvae fed with the two selected strains.

Among the 13 isolates with the higher number of metabolic activities, we selected two strains displaying synergistic and complementing abilities under the perspective to investigate the potential bacteria-mediated metabolic contribution to the holobiont. The two strains belonged to the most represented phylogenetic groups in the collection, i.e., Bacilli and Gamma-proteobacteria. *Bacillus licheniformis* HI169 showed the ability to breakdown cellulose and starch, exhibited uricolytic activity and was able to release ammonia, to dissolve tween 80 and to produce EPS. *Stenotrophomonas maltophilia* HI121, conversely, could digest casein, release ammonia, degrade organic phosphorous, breakdown pectin and had lipase activity (Supplementary Table 1). No inhibition was detected in direct antagonistic plate-assays between the two strains, confirming the possibility to combine them in feeding trials (treatment “*B. licheniformis* HI169 + *S. maltophilia* HI121”). The selected strains were, hence, orally administered, alone or in combination, to BSF 9-day-old larvae reared on a nutritionally poor diet (FD) (Jucker et al., 2017): the larval growth rate and final weight, as well as the prepupal appearance, and pupal weight and length were monitored along the insect development cycle (Figures 3, 4).

Bacterial supplement showed to be significant in determining the larval final weight (ANOVA, F_4_,_10_ = 16; *p* < 0.001). Particularly, pairwise analysis depicted that larvae fed with *B. licheniformis* HI169, *E. coli* DH5α pKan(DsRed) and the bacterial mix gained higher and statistically significant final weights than *S. maltophilia* HI121 addition or the control diet (Supplementary Table 2 and Figure 3A). Regarding the larval growth rate, the statistical analysis unveiled a significant difference considering the treatments HI169, DH5α pKan(DsRed) and control along the time (GAM, Supplementary Table 3).

The pupal weight was statistically influenced by the bacterial treatment (ANOVA, F_4_,_293_ = 7.5; *p* < 0.01), but the pairwise analysis indicated that the administration of the strains HI169, DH5α pKan(DsRed) or the bacterial mix resulted comparable to the control (Figure 4A and Supplementary Table 4). Treatments were also statistically significant in determining the pupal length (ANOVA, F_4_,_295_ = 9.6; *p* < 0.01). Pupal length was maximal with the application of the bacterial mix or strain HI169, which were, however, comparable to the control (Figure 4B and Supplementary Table 5). Regarding the prepupal appearance, we recovered a significant difference considering the treatments (Supplementary Table 6). Statistical analysis showed that the control was significantly different from the other bacterial treatments for which we recorded a delayed prepupal appearance (GAM, Supplementary Table 6 and Figure 4C).

## Discussion

Handling a collection of microbial strains can allow to directly test, by means of *in vitro* and *in vivo* assays, hypotheses about their importance for the insect host, an aspect that is not always possible relying solely on DNA-based techniques (Prosdocimi et al., 2015). Beyond the characterization of their function in the native host, culturable microorganisms can be directly exploited in different biotechnological applications (Crotti et al., 2012; Valiente Moro et al., 2013; Epis et al., 2020). For instance, they have been proposed as probiotics for honeybees to sustain insect health against specific pathogens (Crotti et al., 2013; Daisley et al., 2019) or as beneficial strains to improve the fitness of sterile male insects vs. conventional ones (Ben Ami et al., 2010; Augustinos et al., 2015). Recently, insect symbionts have been suggested as biocontrol agents against phloem-limited pathogens (Iasur-Kruh et al., 2016, 2018; Gonella et al., 2018, 2019; Lidor et al., 2018). In this work, we focused on the bacterial gut inhabitants of the BSF larvae fed on a nutritionally complete diet, on which BSF performance and fitness were previously showed to be higher than on nutritionally poor diets (Jucker et al., 2017). We assumed that the growth performance observed with the nutritionally complete diet should favor the development of a stable and well supporting gut microbiome that may include bacteria with potential probiotic value for BSF.

Our results indicated that the bacterial isolates we obtained from BSF gut encompassed six different classes for a total of 21 genera (Figure 1). As expected, we found an abundance of bacterial members that, as typical inhabitants of the insect digestive tract, have been also specifically retrieved in association with BSF by culture-independent methods (Jeon et al., 2011). Metataxonomic analysis performed on BSF bacterial community showed that members belonging to the four phyla of Proteobacteria, Firmicutes, Bacteroidetes, and Actinobacteria were consistently found in this host (Jeon et al., 2011; Zheng et al., 2013b; Bruno et al., 2019): our approach confirmed the finding of members belonging to these phyla, even if with different proportions, as reasonably expected by the cultivation-based strategy that does not claim to cover the whole bacterial diversity associated to the host, also in consideration that the isolation trials were done a small number of samples. Interestingly and in agreement with previous works (Jeon et al., 2011; Zheng et al., 2013b), we documented the presence of several strains belonging to the genus *Providencia* (Figure 1): Zheng et al. (2013a) have observed that, among others, gravid BSF females were attracted by members of this genus during oviposition. Conversely, we ascertained the presence of a small number of lactic acid bacteria among our isolates, likely due to the inadequacy of the media to support their growth and since media specific for this bacterial group were not used (Wynants et al., 2019).

Within the gut of larvae fed with the nutritional complete diet, the selective and enrichment media allowed the isolation of bacteria capable to produce a wide range of extracellular hydrolytic enzymes (Figure 2). A significant number of isolates presented multiple hydrolytic abilities, traits that potentially confer ecological advantages to the host growing on polymer-rich diets. In some cases, a specific hydrolytic activity was correlated to a specific phylogenetic group, i.e., pectinase activity in *Klebsiella* spp. and *Stenotrophomonas* spp. strains (Supplementary Table 1). The hydrolytic profiles obtained for BSF strains might suggest a primary role of the bacterial partners for the host nutrient supplementation (Engel and Moran, 2013; Gold et al., 2018), specifically contributing to the high levels of lipases and proteases that have been characterized in BSF gut content (Kim et al., 2011b; Bonelli et al., 2019). Taking into account its hydrolytic degradation abilities on complex substrates, BSF could be considered a potential source of enzymes with important industrial applications: recently polymer-degrading enzymes, such as cellulases and serine proteases, have been indeed characterized from *H. illucens* holobiont (Kim et al., 2011a; Lee et al., 2014). However, since strong variations of pH levels, ranging from slightly acidic conditions in the anterior part, to strong acidic values in the middle portion, and to alkaline values in the posterior region, have been reported in BSF midgut (Bonelli et al., 2019; Bruno et al., 2019) and also considering that hypoxic and anoxic conditions might occur in BSF gut (Engel and Moran, 2013; Chouaia et al., 2019), further quantitative verifications of the metabolic activities of the isolates (and of their combination) should be performed under different oxygen concentrations and pH levels, especially regarding the strain *B. licheniformis* HI169, which showed the best performances in *in vivo* tests.

Black soldier fly can be reared on a wide variety of organic substrates which can vary in their nutrient composition (De Smet et al., 2018). For instance, the fruit-based diet used by Jucker et al. (2017), which has been also used in our work, showed a carbon-nitrogen ratio (C/N) of 22.3, suggesting to be a poor source of nitrogenous compounds for the insect. Cammack and Tomberlin (2017) evidenced that a more balanced diet in protein and carbohydrate content allows a faster larval development and a higher survival. For this reason, an “adjunctive” contribution to the nitrogen recycling and uptake provided by gut microbiota could represent a nutritional advantage for the host when grown on an unbalanced diet. Under this perspective, the hydrolytic profiles of our collection revealed that 16.1% of the isolates, mainly belonging to *Ochrobactrum*, *Providencia, Pseudomonas*, and *Stenotrophomonas* genera, showed the ability to degrade uric acid, the main nitrogenous waste compound excreted by Malpighian tubules into the insect anterior hindgut (Supplementary Table S1; Engel and Moran, 2013). Degradation of urea (which could derive, in turn, from uric acid utilization, Martin et al., 2018) with ammonia production was carried out by 32.6% of the strains of our collection, while the majority of the isolates (90.7%) were able to release ammonia from peptone. Protease activity, and specifically the one exerted by serine proteases, was mainly retrieved in the posterior part of the midgut, which reached a pH value of 8.3 (Bonelli et al., 2019). In our collection, 16.6% of the isolates are able to degrade proteins (i.e., milk proteins in our experiments), representing also in this case an extra enzymatic activity source for the insect. Considering a diet with an unbalanced C/N ratio, such as the fruit-based one (which could also represent an organic waste material on which BSF could be reared in a bioeconomy perspective), the gut microbiota might provide the necessary nitrogenous compounds to the host.

Regarding carbohydrate metabolism, several isolates were able to release amylolytic and cellulolytic enzymes. Degradation of carbohydrates (e.g., starch) occurs mainly in the anterior midgut and to a lesser extent in the posterior midgut (Bonelli et al., 2019): this could underline a major role exerted by insect, which led to a small number of active amylolytic and cellulolytic isolates. Conversely, further studies are necessary to characterize cellulolytic bacteria in the insect. In conclusion, as a matter of fact we observed that few strains showed to be active on complex substrates (e.g., cellulose or starch), likely sustaining other gut symbionts which could degrade simple substrates (e.g., degradation of peptone).

Finally, in our survey we found a widespread presence of phytase activity among bacterial isolates obtained from larvae fed on the standard diet, a nutritional source that can contain phytate (Magallanes-López et al., 2017; Kaplan et al., 2019). Presence of phytate in feedstuffs could reduce the availability of essential minerals, amino acids and proteins (Wodzinski and Ullah, 1996) and its degradation could release phosphate that could be available for the host or other members of the gut microbiota. Beyond its primary function as phosphorous and energy storage, phytic acid in plant tissues also plays a defensive role against phytophagous insects: Green et al. (2001) showed in fact a positive correlation between the presence of phytic acid in the diet and the mortality of three Lepidoptera species. Moreover, due to the increasing exploitation of *H. illucens* to reduce food or agricultural wastes, the degradation of phytic acid by the gut microbial community could be of interest to improve the insect growth rate and physiological status. While different studies have evaluated the positive effect derived from the supplementation of microbial phytase to the diet of broilers chickens and pigs (Dersjant-Li et al., 2013), there is still poor information about the influence of phytate on the insect growth and development, a topic that could be of pivotal importance for the emerging insect farming practices.

Under the perspective of BSF as a sustainable alternative of feed or fuel (Diener et al., 2009; Zheng et al., 2012b; Nguyen et al., 2015), different works are currently devoted to the optimization of its rearing conditions in order to obtain high amounts of insect biomass reared on low quality feed material represented by waste (Diener et al., 2009; Zheng et al., 2012b). Nevertheless, despite the well-known importance of the host-associated microbiota, few efforts have been made so far to evaluate if bacterial companions can boost the insect development and biomass gaining, also considering nutritionally unbalanced rearing conditions (Yu et al., 2011; Zheng et al., 2012a; Xiao et al., 2018; Mazza et al., 2020). In particular, addition of BSF companion bacteria such as *Bacillus* spp. strains to chicken manure resulted in a co-conversion process that shortened the manure processing and the insect developmental times, enhanced the insect biomass yield and influenced BSF nutrient accumulation (Yu et al., 2011; Xiao et al., 2018; Mazza et al., 2020). In our work we also found a positive influence on the insect performance following the administration of BSF-associated bacteria: in particular, the addition of strain *B. licheniformis* HI169 allowed an increase of the larval weight when compared to the control (Figure 3). Also the larval growth rate resulted higher when *B. licheniformis* HI169 was added to the diet in comparison to the control (Figure 3). It is noteworthy to mention that, however, following the bacterial administrations, larvae needed longer time to pupate than the control ones (Figure 4). Pupal weight and length were maximal with the application of the strain HI169 alone or in combination with *S. maltophilia* HI121, but they were, however, comparable to the control (Figure 4). Conversely, larvae and pupae fed on the diet supplemented with *S. maltophilia* HI121 did not differ from the control ones (Figures 3, 4), but differed from larvae and pupae reared on the diet supplemented with *B. licheniformis* HI169 alone or in combination. We also detected a significant improvement of the larval weight following the administration of the outsider control strain *E. coli*. This could lead to hypothesize that the addition of specific bacteria may affect the nutritional quality of the insect unbalanced diet, through a supplementation of microbial proteins or cofactors, partially explaining why we observed good performances for *E. coli* DH5α pKan(DsRed) in relation to the larval weight. Otherwise, the differential promotion effect given by *B. licheniformis* HI169 could be attributed to specific activities given to the host by this strain. The hydrolytic screenings of *B. licheniformis* HI169 revealed its ability to degrade complex compounds such as cellulose, starch and uric acid, to release esterase enzymes that degrade organic molecules and to produce EPS by which the bacterium can adhere to the surfaces, e.g., intestinal epithelium. However, a further confirmation aimed to experimentally verify that the metabolic activities can take place *in vivo* should be provided. These behaviors, especially if compared to the one exerted by *S. maltophilia* HI121, underline the need to evaluate directly the effect of the bacterial administrations on the insect performance. Finally, these data underlined the positive effect of specific bacterial administrations on the insect performance: to strengthen the outcome of the experiments here described (which were run with three replicates, following the set-up and methods already established in other works, e.g., Rehman et al., 2017; Lalander et al., 2019), it could be interesting to consider different time points to observe a consistency of our findings also in consideration that the observed performance improvement was minimal. Furthermore, it could be relevant to measure the abundance of the beneficial bacteria in the insect gut to understand their colonization ability of the host.

Selection of beneficial microorganisms with a positive impact on the host development and growth might be meaningful also in economic terms. Probiotic microorganisms are known to produce antimicrobial compounds to counteract pathogens, to stimulate the host immune system, to affect the dynamics of the gut microbial populations, to increase the digestion and absorption of nutrients and to prevent pathogens colonization (Grau et al., 2017). Application of beneficial strains in insect farming could lead to the enhancement of the host resistance, preventing the spread of pathogens in the insect rearing, and thus reducing/avoiding the cost and use of antibiotic treatments to manage disease outbreaks. Improved growth performance by the administration of beneficial strains could permit the utilization of disposal material as insect rearing substrate, leading to a valorization of the waste also from the economic point of view, reducing the cost of diet ingredients under the perspective of circular economy principles. A life cycle assessment (LCA) analysis would be thus useful to evaluate the environmental aspects and potential impacts associated with this microbe-assisted strategy in insect farming (Iribarren et al., 2012).

## Conclusion

Gut symbionts’ manipulation is a promising field of research aimed to optimize the waste disposal via insect feeding, e.g., using BSF, especially under the perspective to obtain a sustainable alternative of feed or fuel (van Huis, 2013). Under this perspective culture-dependent techniques allow to screen the culturable fraction of the host microbiota to seek for key bacteria in host nutrition and physiology.

The bacterial collection obtained from BSF larval gut was mainly constituted by members of Gamma-proteobacteria and Bacilli classes. The hydrolytic profiles of the isolates revealed their potential contribution to the host nutrition in terms of carbon, nitrogen and phosphorous recycling and uptake and allowed the selection of candidates that were further used in *in vivo* trials with the host. Our analysis supports the possibility to exploit BSF-associated strains to enhance the insect growth performance when reared on an unbalanced nutritionally poor diet (Jucker et al., 2017) that can simulate an insect rearing substrate in a bioeconomy perspective. We identified *B. licheniformis* HI169 as one of the bacteria able to amend the physiology and performance of the insect growth, highlighting the need of bacterial administration trials to provide feasible microbial solutions to improve the host growth. Furthermore, the collection of BSF-associated bacterial strains is available for future studies of insect feeding in order to evaluate the host performances when reared on different organic substrates.

## Data Availability Statement

The datasets presented in this study can be found in online repositories. The names of the repository/repositories and accession number(s) can be found at: https://www.ebi.ac.uk/ena, PRJEB30516.

## Author Contributions

EC and SS designed the study. MC, CJ, ML, and SS carried out the experiments. MC, CJ, ML, MF, SS, and EC analyzed the data. EC, SS, SB, and DD supported the research. MC and EC wrote the first draft of the manuscript. All authors contributed to the manuscript revision, read and approved the submitted version.

## Conflict of Interest

The authors declare that the research was conducted in the absence of any commercial or financial relationships that could be construed as a potential conflict of interest.
