# Supplementary material for: Hydrolytic Profile of the Culturable Gut Bacterial Community Associated With Hermetia illucens
Source: Front Microbiol. 2020 Aug 12;11:1965. doi: 10.3389/fmicb.2020.01965 (PMC7434986; doi:10.3389/fmicb.2020.01965)
Supplement: Supplementary file 2 [file Data_Sheet_1.PDF]

Supplementary Material

Hydrolytic profile of the culturable gut bacterial community associated with *Hermetia illucens*

Matteo Callegari[1,2], Costanza Jucker[1], Marco Fusi[2,3], Maria Giovanna Leonardi[1], Daniele Daffonchio[2], Sara Borin[1], Sara Savoldelli[1], Elena Crotti[1,*]

[1]Dipartimento di Scienze per gli Alimenti, la Nutrizione e l'Ambiente (DeFENS), Università degli Studi di Milano, via Celoria 2, 20133 Milano, Italy

[2]Red Sea Research Center (RSRC), King Abdullah University of Science and Technology (KAUST), Thuwal 23955-6900, Saudi Arabia

[3]School of Applied Sciences, Edinburgh Napier University, Edinburgh, UK

Correspondence:
Dr. Elena Crotti
elena.crotti@unimi.it

Supplementary Table 1. Taxonomic affiliation, isolation source and metabolic profiles of the bacterial isolates (n=193) obtained from BSF larvae reared on SD. NA-UA=nutrient agar-uric acid medium; Amyl.=amylolytic activity; Cell.=cellulolytic activity; Pectin=pectinase activity; Est.=esterase activity; Lip.=lipase activity; Prot.=protease activity; NH_3=ammonia production; Urease= urease activity; Uric acid=uricolytic activity; Phytase=phytase activity; C=pathways related to carbon metabolism; Fat= pathways related to fat metabolism; Protein= pathways related to protein metabolism; N=pathways related to nitrogen metabolism; P=pathway related to phosphorous metabolism; N° activities=refers to the sum of the hydrolytic activities without considering EPS production; Ad.=adhesion. Isolates (n=13) with the highest scores are highlighted in light blue.

Supplementary Table 2. ANOVA pairwise test performed on the datasets related to the larval final weight. Asterisks indicate statistical significance (*p<0.05; **p<0.01; ***p<0.001).

Treatment	P adj	
DH5α-Control	0.0019881	**
HI121-Control	0.9854966	
HI169-Control	0.0014622	**
HI169+HI121-Control	0.0034544	**
HI121-DH5α	0.0040090	**
HI169-DH5α	0.9993115	
HI169+HI121-DH5α	0.9939722	
HI169-HI121	0.0029083	**
HI169+HI121-HI121	0.0071301	**
HI169-HI169+HI121	0.9681001	

P adj: corrected *p*-value with Bonferroni correction.

Supplementary Table 3. Generalize Additive Model on the dataset related to the larval growth rate. Asterisks indicate statistical significance (*p<0.05; **p<0.01; ***p<0.001).

Treatment	Estimate	Std. Error	t value	Pr(>|t|)	
Control	0.94774	0.01543	61.408	< 2e-16	***
DH5α	0.04618	0.02155	2.143	0.03284	*
HI121	-0.03205	0.02148	-1.492	0.13665	
HI169	0.05567	0.02133	2.610	0.00945	**
HI169+HI121	-0.01369	0.02130	-0.643	0.52068	
Approximate significance of smooth terms	edf	Ref.df	F	P value	
Days	7.883	8.612	864.4	<2e-16	***

Estimate: Slope of the line; Std. Error: Standard Error; t value: t-test statistic; Pr(>|t|): *p* statistic; edf: estimated degree of freedom; Ref.df: Residual estimate degree of freedom; F: F statistic; P value: *p* statistic.

Supplementary Table 4. ANOVA pairwise test performed on the datasets related to pupal weight. Asterisks indicate statistical significance (*p<0.05; **p<0.01; ***p<0.001).

Treatment	P value	
DH5α-Control	0.9884	
HI121-Control	0.1673	
HI169-Control	0.2063	
HI169+HI121-Control	0.4091	
HI121-DH5α	0.3194	
HI169-DH5α	0.1024	
HI169+HI121-DH5α	0.2198	
HI169-HI121	0.0047	**
HI169+HI121-HI121	0.0101	*
HI169-HI169+HI121	0.9837	

Supplementary Table 5. ANOVA pairwise test performed on the datasets related to pupal length. Asterisks indicate statistical significance (*p<0.05; **p<0.01; ***p<0.001).

Treatment	P value	
DH5α-Control	0.8654	
HI121-Control	0.1591	
HI169-Control	0.0878	
HI169+HI121-Control	0.3681	
HI121-DH5α	0.5467	
HI169-DH5α	0.0198	*
HI169+HI121-DH5α	0.0942	
HI169-HI121	0.0020	**
HI169+HI121-HI121	0.0085	**
HI169-HI169+HI121	0.8456	

Supplementary Table 6. Generalize Additive Model on the dataset related to the appearance of prepupae. Asterisks indicate statistical significance (*p<0.05; **p<0.01; ***p<0.001).

Treatment	Estimate	Std. Error	t value	Pr(>|t|)	
Control	0.089067	0.001634	54.511	< 2e-16	***
DH5α	-0.009633	0.002311	-4.169	4.04e-05	***
HI121	-0.014600	0.002311	-6.318	9.90e-10	***
HI169	-0.014683	0.002311	-6.355	8.06e-10	***
HI169+HI121	-0.030167	0.002311	-13.055	< 2e-16	***
Approximate significance of smooth terms	edf	Ref.df	F	P value	
Days	4.52	5.541	658.7	<2e-16	***

Estimate: Slope of the line; Std. Error: Standard Error; t value: t-test statistic; Pr(>|t|): *p* statistic; edf: estimated degree of freedom; Ref.df: Residual estimate degree of freedom; F: F statistic; P value: *p* statistic.
